# Promising Roles of Exosomal microRNAs in Systemic Lupus Erythematosus

**DOI:** 10.3389/fimmu.2021.757096

**Published:** 2021-12-13

**Authors:** Wenqian Wang, Chenran Yue, Sheng Gao, Shuting Li, Jianan Zhou, Jiaqing Chen, Jiahong Fu, Weijian Sun, Chunyan Hua

**Affiliations:** ^1^ Department of Surgery, The Second Affiliated Hospital and Yuying Children’s Hospital of Wenzhou Medical University, Wenzhou, China; ^2^ School of Basic Medical Sciences, Wenzhou Medical University, Wenzhou, China; ^3^ Laboratory Animal Center, Wenzhou Medical University, Wenzhou, China; ^4^ School of the 2nd Clinical Medical Sciences, Wenzhou Medical University, Wenzhou, China

**Keywords:** systemic lupus erythematosus, exosomal microRNA, immunomodulation, Toll-like receptor, biomarker, therapy

## Abstract

Systemic lupus erythematosus (SLE) is a prototypic autoimmune disease characterized by the loss of immune tolerance. Lupus nephritis (LN) is still a major cause of the morbidity and mortality of SLE. In clinical practice, diagnosis, and therapy of SLE is complicated and challenging due to lack of ideal biomarkers. Exosomes could be detected from numerous kinds of biological fluids and their specific contents are considered as hallmarks of autoimmune diseases. The exosomal miRNA profiles of SLE/LN patients significantly differ from those of the healthy controls making them as attractive biomarkers for renal injury. Exosomes are considered as optimal delivery vehicles owing to their higher stable, minimal toxicity, lower immunogenicity features and specific target effects. Endogenous miRNAs can be functionally transferred by exosomes from donor cells to recipient cells, displaying their immunomodulatory effects. In addition, it has been confirmed that exosomal miRNAs could directly interact with Toll-like receptors (TLRs) signaling pathways to regulate NF-κB activation and the secretion of inflammatory cytokines. The present Review mainly focuses on the immunomodulatory effects of exosomal-miRNAs, the complex interplay between exosomes, miRNAs and TLR signaling pathways, and how the exosomal-miRNAs can become non-invasive diagnostic molecules and potential therapeutic strategies for the management of SLE.

## Introduction

Systemic lupus erythematosus (SLE) is a prototypic autoimmune disease which is characterized by the loss of immunological tolerance, production of inflammatory cytokines and autoantibodies that form immune complex (IC) deposits (1). IC deposition is a major event in the renal glomeruli of patients with lupus nephritis (LN) which is one of the most serious manifestations of SLE and is associated with significant morbidity and mortality (2). To date, our understanding of SLE pathogenesis has increased. However, SLE patients are still suffering from a meaningful burden of poor outcomes (3). Great effort is required to define easily-measurable, highly-sensitive, non-invasive, and reliable biomarkers for SLE in order to early diagnosis and assess disease activity or improve therapeutic regimen in clinical application (4).

MicroRNAs (miRNAs) are a class of small non-coding RNA (about 22 nucleotides) that play a critical role in the regulation of diverse biological processes. Altered miRNA profile is closely associated with progression or remission of SLE (5–7). miRNAs are not only localized within the cell but presence in various biological fluids. A majority of extracellular miRNAs detected in human urine, serum, and saliva are concentrated in exosomes and they are protected from degradation by RNases through inclusion in a lipid bilayer (8, 9).

Exosomes are a type of extracellular vesicles (EVs), ranging from 30 to 150 nm in diameter. Existing evidence suggests that the levels of circulating exosomes correlate with disease activity in patients with SLE (10). Exosome-delivered miRNAs carry out transport of genetic information, and can be taken as diagnostic biosignatures or therapeutic approaches for autoimmune diseases. Abnormal expression of exosomal-miRNAs has been found in SLE/LN patients and this exosomal-miRNA profile could reflect the SLE activity and histological alterations (11–13). These findings highlight the promising applications of exosomal-miRNAs as ideal biomarkers of SLE progression, especially the kidney activity.

Increasing evidence indicate that Toll-like receptors (TLRs) play critical roles in SLE pathogenesis (14). There is much interest in targeting TLRs for the prevention and treatment of SLE (15). Specifically, miRNAs within exosomes could be transferred to recipient cells, where they could canonically bind to their target mRNAs or directly interact with TLRs (16, 17). Nevertheless, effects of exosomal miRNAs by targeting TLRs pathways on the development of SLE are not well reviewed until now. This review mainly introduce the characteristics of exosomes, the mechanisms of sorting miRNAs into exosomes, and specifically focus on immunomodulatory properties of exosomal miRNAs. Moreover, the interplay between exosomes, miRNAs and TLRs are highlighted. The potential applications of exosomal miRNAs as diagnostic biomarkers and therapeutic strategies for SLE in clinical settings are also discussed. This review will renew our understanding of the mechanisms underlying intracellular communication and provide an attractive therapeutic approach for autoimmune diseases.

## Characteristics of Exosomes

### Biogenesis of Exosomes

**Figure 1:**
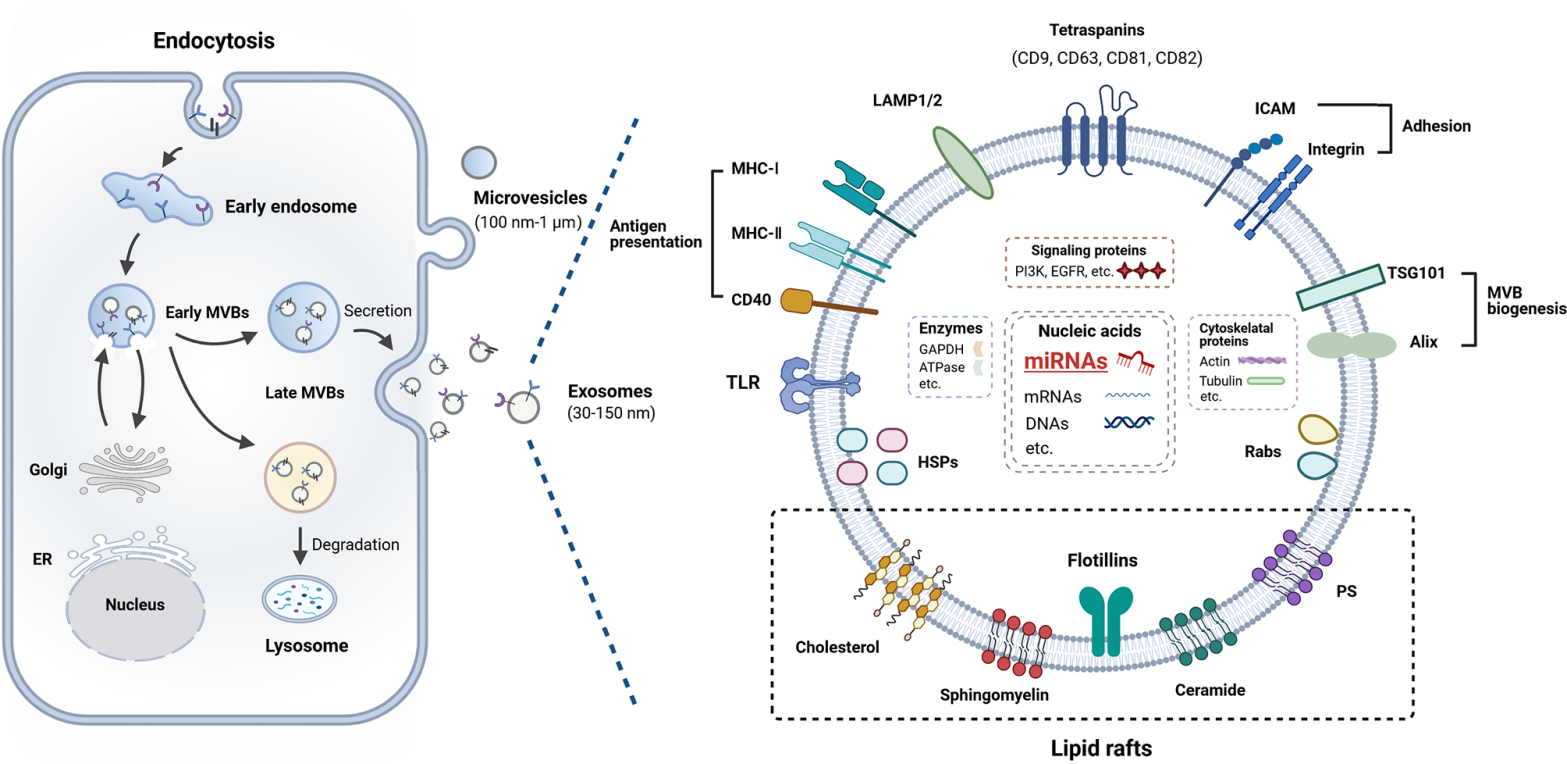
Biogenesis and composition of exosome. Exosomes are derived from endosomes that formed from cellular membrane compartments. The endosomes harboring intraluminal vesicles (ILVs) are called multivesicular bodies (MVBs). During this process, early endosomes (EEs) communicate with the Golgi apparatus through bidirectional vesicle exchange. MVBs may be degraded by lysosomes or fuse with the plasma membrane, and then release ILVs into extracellular space termed as exosomes. Exosomes contain a range of proteins, miRNAs, mRNAs, and DNA molecular cargo. Details are provided in the main text.

Exosomes are released by most cell types into the extracellular space (18) and they can be found in various body fluids (19). The mechanism of the biogenesis of exosomes is very complex (**Figure 1**). In the first stage, inward invagination of clathrin-coated microdomains on plasma membrane forms the endocytic vesicles, creating early endosomes (EEs) that can interact with the Golgi apparatus and the endoplasmic reticulum, then these early EEs matured into late endosomes (20). Exosomes are intraluminal vesicles (ILVs) generated within endosomal system by inward budding of endosomes to form multivesicular bodies (MVBs) (21). Exosome-specific proteins and other biomolecules are sorted into the ILVs. The endosomal sorting complex responsible for transport (ESCRT) complex facilitates the development of invaginated vacuoles into EEs and participates in the recognition of ubiquitinated cargo by MVBs, as well as the invagination of the MVBs outer membrane (21). In last stage, later MVBs can be transformed in one of two ways: degradative MVBs or secretory MVBs. Degradative MVBs fuse with lysosome and their contents will undergo lysosomal degradation. Secretory MVBs fuse with the plasma membrane and release ILVs and these released ILVs are termed as exosomes (20–22).

Determination of the destination of these vesicles is not fully understood and they were demonstrated to be mediated by Rab family of proteins (23). Exosomes may exert their function in distinct modes: 1) triggering a second messenger signaling; 2) internalization into recipient cells and downloading their contents; and 3) releasing its components into the extracellular space (24).

### Composition of Exosomes

The biogenesis of exosomes determines their composition which includes certain extracellular, membrane and endosomal-associated components from their origin, including a variety of proteins, lipids, RNAs, DNAs, et al., termed as “cargo” (25, 26). Many research teams have focused on the contents of exosomes, which leads to the knowledge of their composition has grown significantly (**Figure 1**). Specific database ExoCarta (http://www.exocarta.org) lists the molecules that have been found in exosomes.

Although the composition of exosomes shows variation, exosomes usually harbor cytoskeleton proteins, metabolic enzymes, signal transduction proteins, heat shock proteins, proteins involved in MVB biogenesis (Alix, TSG101, and clathrin), lysosomal-associated membrane proteins (LAMP1 and LAMP2), tetraspanins (CD81, CD9, CD63, and CD82), adhesion molecules (ICAMs and integrins), Rab proteins, as well as antigen presentation related proteins (CD86, MHC-I and -II) (20, 25, 27, 28). These proteins can be used as biomarkers to detect the presence of exosomes.

The main lipid compounds of the membrane of exosomes include cholesterol, sphingolipids, and glycerophospholipids (20, 29). Previous studies have also reported that exosomes contain bioactive lipids, such as prostaglandins and leukotrienes (30). Moreover, exosomes also harbor a wide array of RNAs in the form mRNA, miRNA, transfer RNA (tRNA), ribosomal RNA (rRNA), piwi-interacting RNAs (25, 31). A specific database (http://www.exoRBase.org), exoRBase, is a repository of different kinds of RNAs from RNA-seq data analyses of human blood exosomes, and experimental validations from the published literature (32). It has also been investigated that exosomes contain saccharide groups on their surface membranes including mannose, polylactosamine, α-2,6 sialic acid, and complex N-linked glycans (33).

## Mechanisms for Sorting of miRNAs Into Exosomes

Several studies have shed light on the mechanisms for sorting of miRNAs into exosomes. According to previous literature, the process of sorting miRNAs into exosomes follows distinct proposed ways. Potential modes are shown as follows: 1) The sumoylated heterogeneous nuclear ribonucleoprotein-(hnRNP-) dependent pathway which mainly includes hnRNPA2B1 and hnRNP-Q: miRNAs that contain the EXO motif (GGAG motif in the the 3′portion of miRNA sequences) can be recognized by sumoylated hnRNPA2B1 (34). Once this recognition occurs, specific miRNAs are efficiently sorted into exosomes, while miRNAs without this motif remain in the cytoplasm (34). miRNAs that contain the hEXO motif are recognized by hnRNP-Q also called SYNCRIP, leading to sorting of these miRNAs into the exosomes (35). 2) Y-box protein 1 (YBX1) is also involved in sorting miRNAs (36). In HEK293T cells, YBX1 was identified as a protein required for miR-223 sorting into the exosomes (36). Besides the sorting of miRNAs, YBX1 is also involved in the sorting of other non-coding RNAs in exosomes (37). 3) The involvement of neutral sphingomyelinase 2-(nSMase2-) in exosomal miRNA sorting mechanism: after the inhibition of nSMase2 expression and function, the quantum of exosomal miRNAs reduces, which indicates the role of nSMase2 in miRNA package into exosomes (38). 4) The miRNA-induced silencing complex-(miRISC-) dependent pathway which relies on Ago2: combination of Ago2 and miRNA to form Ago2-miRNA complex is packaged into exosomes which is regulated by MEK/ERK pathway (39, 40). After the deficiency of Ago2, the main component of miRISC is more conducive to bind to the 5′-end of miRNA, reducing the number of privileged-transported miRNAs in exosomes (41). 5) Post-translational modification related pathway: Koppers-Lalic and his colleagues reported that uridylation of 3′-ends of endogenous miRNAs promotes their sorting to exosomes (42).

## Immunomodulatory Effects of Exosomal miRNAs

Not only intracellular miRNAs have been recognized as key modulators in gene expressions (43), exosome-shuttled miRNAs have also been revealed to possess pivotal immunomodulatory properties. The transfer of miRNAs through exosomes plays important roles in the physiological immune responses and also in the development of autoimmune diseases. The immunologic function of exosomal miRNAs-derived from immune cells is mainly discussed in this section (**Table 1**).

**Table 1:** Functions of exosome-associated miRNAs in immune modulation.

Source		Exo-miRNA	Recipient		Function	Ref.
Species	Cell		Species	Cell/Animal		
Human	UC-MSC	miR-146a	Mouse	Mø	Enhancing polarization of macrophages to M2 and attenuating inflammation	(44)
Rat	BM-MSC	miR-146a	Rat	Colitis	Attenuating inflammation in colitis of rats *via* NF-κB	(45)
Human	UC-MSC	let-7b	Human/Rat	Mø/diabetic rat	Modifying macrophage polarization and alleviating chronic inflammation	(46)
Mouse	MSC	miR-21	Mouse	Mø/sepsis	Inducing macrophage M2 polarization and ameliorate sepsis	(47)
Mouse	BM-MSC	miR-150-5p	Human Mouse	FLS/CIA	Decrease migration and invasion in FLS and reducing joint destruction in CIA mouse model	(48)
Rat	BM-MSC	miR-192-5p	Rat	CIA	Delay the inflammatory response in CIA model	(49)
Human	UC-MSC	miR-181a	Mouse	I/R injury	Possessing the immune-suppressing role and exerting a therapeutic effect on I/R injury	(50)
Human	UC-MSC	miR-181c	Rat	Mø/burn model	Attenuating burn-induced inflammation by inhibiting TLR4 pathway	(51)
Mouse	DC	miR-148a miR-451, et al.	Mouse	DC	An inhibitory effect on target mRNAs of acceptor DC	(52)
Mouse	BM-DC	miR-155 miR-146	Mouse	BM-DC	miR-155 enhancing while miR-146a reducing inflammatory gene expression	(53)
Mouse	BM-DC	miR-146	Mouse	EAMG	Suppressing ongoing clinical MG in mice and altering Th cell profiles	(54)
Mouse	BM-DC	miR-682	Mouse	Renal allograft model	Promoting Tregs differentiation to induce immune tolerance after kidney transplantation	(55)
Human	M1	miR-16-5p	Human	GC cell	Enhancing T cell immune response by regulating PD-L1 in GC	(56)
Human	Mø	miR-223	Human	Monocytes	Inducing the differentiation of recipient monocytes	(57)
Human	TAM/ M2	miR-29a-3p miR-21-5p	Human	CD4^+^ T cell	Inducing the Treg/Th17 cell imbalance in EOC	(58)
Mouse	Mø	miR-155	Mouse	Cardiac fibroblasts	As a regulator for fibroblast proliferation and inflammation	(59)
Mouse	Mø	miR-21-3p miR-146a miR-146b	Mouse	Pain model	Reducing paw swelling and relieving inflammatory response	(60)
Mouse	B cell	anti-miR-150	Mouse	CD8^+^ T cell	Down-regulation of endogenous miR-150	(61)
Mouse	B cell	anti-miR-155	Mouse	Mø	Reduction in LPS-stimulated TNF-α production	(62)
Human	B cell	miR-155	Human	Hepatocytes	Inhibition of HCV replication in hepatocytes from RA patients	(63)
Mouse	CD8^+^ Ts cell	miR-150	Mouse	Te cell	Inhibition of the contact sensitivity of Te cell	(64)
Mouse	CD8^+^ Ts cell	miR-150	Mouse	Mø	Modulation of Mφ antigen-presenting function	(65)
Human	T cell	miR-142-3p	Human	Glandular cell	Impairment of the function of salivary gland epithelial cells	(66)
Human Mouse	T cell	miR-142-3p miR-142-5p miR-155	Mouse	Pancreatic β cell/NOD mice	Promoting pancreatic β cell death and contributing to T1D development	(67)
Mouse	CD4^+^ T cell	miR-155-3p miR-25-3p miR-20a-5p	Mouse	B cell	Control germinal center reaction and antibody production	(68)
Human	T cell line	miR-335	Human	APC	Down-regulation of the target gene expression in APC	(69)
Mouse	Treg cell	let-7d	Mouse	Th1 cell	Suppression of Th1 cell proliferation and cytokine secretion	(70)

Exo, exosome; miR, microRNA; UC-MSC, umbilical cord-derived MSC; Mø, macrophage; BM-MSC, bone marrow-derived MSC; NF-κB, nuclear factor-κB; FLS, fibroblast-like synoviocyte; CIA, collagen-induced arthritis; I/R, ischemia-reperfusion; TLR4, Toll-like receptor 4; DC, dendritic cell; BM-DC, bone marrow-derived DC; MG, myasthenia gravis; EAMG, experimental autoimmune MG; Th, helper T cell; Treg, regulatory T cell; PD-L1, programmed cell death-ligand 1; TAM, tumor-associated macrophage; EOC, epithelial ovarian cancer; GC, gastric cancer; HCV, hepatitis C virus; RA, rheumatoid arthritis; Ts, suppressor T cell; Te, effector T cell; NOD, non-obese diabetic; T1D, type 1 diabetes; APC, antigen-presenting cell.

### MSC-Derived Exosomal miRNAs

Mesenchymal stem cells (MSCs) have been proposed for the therapy of autoimmune diseases for their immunosuppressive properties which is closely associated with the exosomes. miR-146a acts as a critical molecular brake on aberrant inflammation which is the root cause of numerous human diseases (71). Therefore, it is well known for its anti-inflammatory effects in several kinds of autoimmune disorders such as rheumatoid arthritis (RA) (72), SLE (73), and psoriasis (74). Mice loss of miR-146a develop a spontaneous autoimmune disorder later in life and many of them die prematurely (75). Exosomes are often used as delivery tools to study the therapeutic effect of miR-146a in a variety of inflammatory diseases *in vivo*. Pre-treatment with IL-1β strongly up-regulated the level of miR-146a in human umbilical cord-derived MSCs (UC-MSCs), and this miRNA was selectively packaged into exosomes (44). Furthermore, exosome-mediated transfer of miR-146a contributed to the enhanced immunomodulatory properties of MSCs by ameliorating the symptoms of murine sepsis and inducing macrophage polarization toward M2 phenotype (44). Administration of EVs-miR-146a generated from MSCs dampened TRAF6 and IRAK1 expression, and suppressed TNBC-induced inflammatory cytokines production in colon tissue of rats through inhibiting NF-κB signaling pathway (45).

LPS-preconditioned MSCs has been an attractive therapeutic approach for chronic diseases and tissue injury as demonstrated by Ti et al. They found that exosomes from LPS-preconditioned MSCs possessed advantages for the transform of macrophages into M2 type. This effect was mainly mediated by let-7b that shuttled by MSC-exosomes. *In vivo*, LPS pre-exosomes greatly alleviated inflammation (46). Exosomes isolated from IL-1β primed MSC could induce M2-polarization of macrophages and attenuate the symptoms in septic mice by delivery of miR-21 (47).

In RA patients, levels of miR-150-5p in serum and synovial tissues were strongly decreased compared with osteoarthritis patients. *In vitro*, miR-150-5p was effectively transferred by MSC-derived exosomes to FLS, and reversed the migration and invasion of FLS by directly suppressing the expression of its target genes MMP14 and VEGF (48). *In vivo*, administration of MSC generated exosomal-miR-150-5p alleviated joint inflammation in collagen-induced arthritis (CIA) mouse model (48). Additionally, miR-192-5p overexpressed exosomes derived from BM-MSC could repress the levels of inflammatory cytokines in synovial tissues and serum of CIA rats (49).

miR-181 and miR-21 could also be selectively packaged into exosomes and act as efficient rheostat to modulate inflammatory response. Studies demonstrated that miR-181a delivery by MSC-exosome downregulated TNF-α and IL-6, as well as increased the expression of the IL-10 in PBMCs (50). Previous study found that burn injury significantly increased the inflammatory reaction induced by LPS. Whereas, human UC-MSC derived exosomes enriched miR-181c effectively suppressed LPS-stimulated inflammatory response in macrophage (51). These data suggest that miRNAs shuttled by MSC-derived exosomes possess complex pleiotropic effects on different aspects of inflammation.

### DC-Derived Exosomal miRNAs

Dendritic cells (DCs) are the most important antigen presenting cells (APCs) in immune system. miR-146a functions as a negative master regulator for DCs maturation and activation. Previous studies demonstrated that DCs efficiently transfer endogenous exosomal-miRNAs including miR-146a and miR-155 to other target DCs and they are internalized, and fused with recipient DCs (52, 53). Exosomal miR-146a inhibits the endotoxin-induced inflammation, whereas exosomal miR-155 promotes inflammation to the same stimulus in recipient DCs (53). These studies represent a novel mechanism of DC-DC communication.

Myasthenia gravis (MG) is a neurological autoimmune disease, resulting from aberrant activation of T and B lymphocytes in the immune system. Exosomes from miR-146a transfected DCs were anti-inflammatory and they expressed lower levels of CD80 and CD86 than that of exosomes from control DCs. Exosomes rich in miR-146a could alleviate clinical symptoms of experimental autoimmune MG mice, and this therapeutic effects were antigen-specific (54). Moreover, they could suppress T lymphocyte proliferation and shift T helper (Th) cell profiles from Th1/Th17 to Th2/Treg subsets (54).

The potential of DC-derived exosomes to trigger immunity or tolerance depends on the maturation status or subtypes of DCs. Studies have reported that exosomes from immature DCs (imDCs) or regulatory DCs have shown certain therapeutic prospects in autoimmune disease by inducing T-cell tolerance. Pang et al. demonstrated that miR-682 was enriched in imDCs secreted exosomes (imDex) which remarkably increased survival rate, decrease rejection associated cytokines (IFN-γ, IL-2, and IL-17) production in renal allograft model mice (55). Moreover, imDex shuttled miR-682 suppressed the IL-17^+^CD4^+^ T cells and promoted Tregs differentiation (55) playing an important role in autoimmunity.

### Macrophage-Derived Exosomal miRNAs

Macrophages, another kind of APCs, can also produce exosomes with immunomodulatory function. Blockade of PD1/PD-L1 checkpoints could lead to T cells activation and inhibit gastric cancer (GC) proliferation. M1 macrophage-secreted exosomes carrying miR-16-5p were confirmed to trigger T cell response which inhibited tumor progression by targeting PD-L1 in GC cells (56).

Ismail et al. found that exosomel miR-223 contained in the macrophage were transported to and activated the target cells (57). A Treg/Th17 cell imbalance characterizes many human diseases, including systemic lupus, diabetes, and multiple kinds of cancers. Tumor-associated macrophage (TAM) derived exosomes transfer miRNAs to induce the Treg/Th17 imbalance by targeting STAT3 in CD4^+^ T cells in epithelial ovarian cancer (EOC) (58). These results revealed a exosome-related mechanism of TAMs in tumor progression supplied a therapeutic strategy in EOC.

miR-155 expression was enriched in activated macrophages derived exosomes. These miR-155 containing exosomes inhibited cardiac fibroblast proliferation and promoted inflammation (59). It was demonstrated that exosomes derived from LPS-stimulated macrophages carry higher levels of 3 murine homologs for human miRNAs (60). Additionally, these exosomes were able to reduce paw swelling and relieve inflammatory response in complete Freund’s adjuvant-induced pain model (60). Based on this observation, we can summarize that macrophage-derived exosomes communicate with a variety cell types, suggesting the impact of these microvesicles may be widespread.

### B Cell-Derived Exosomal miRNAs

Primary B lymphocytes can be genetically programmed with plasmid DNA comprising the coding sequence for anti-miR-150. Intriguingly, anti-miR-150 synthesized in these engineered B cells were secreted both as free and EV-packed fractions while only EVs-carried ones were apparently internalized by CD8^+^ T cells during antigen-mediated activation (61). This implies that B cells represent an efficient platform for the synthesis and delivery of noncoding RNAs to regulate adaptive immunity, paving the way for miRNA-based therapy.

miR-155 exerts a positive regulation in LPS-induced TNF-α production by macrophages through enhancing its translation (76). Given the importance of macrophages in multiple inflammatory diseases, including autoimmune diseases, exosomes might be used as vehicles to deliver a miR-155 inhibitor to alleviate inflammatory responses. In this sense, Momen et al. generated B cell derived exosomes to deliver exogenous miR-155 inhibitor into macrophages. They found that treatment of macrophage cell lines with miR-155 inhibitor loaded exosomes inhibited the endogenous miR-155 level in recipient cells and significantly reduced TNF-α production (62). miR-155 was demonstrated to promotes the expression of inflammatory cytokines, and it is associated with HCV production inhibition (77, 78). Exo-miR-155 levels were increased in RA patients infected with HCV. *In vitro* study showed that miR-155 delivered by B cell-derived exosomes could inhibit HCV replication in hepatocytes (63).

### T Cell-Derived Exosomal miRNAs

Bryniarski et al. firstly presented evidence that CD8^+^ suppressor T-cell (Ts) population produced the exosome-like nanovesicles carried inhibitory miR-150 that specifically target the effector T-cell mixture of contact sensitivity (CS) (64). It provides translational possibilities for the treatment of several human diseases. In murine CS reaction, antigen-specific immune tolerance is also mediated by exosomes derived by CD8^+^ Ts cells. These exosomes were coated with antibody and carried miR-150 that were formerly suggested to suppress CS. Nazimek et al. demonstrated the essential role of Ts cell-secreted exosomes in antigen-specific immune suppression (65).

miR-142-3p has been previously documented to express in the salivary glands of patients with Sjogren’s syndrome (SS), but not in healthy controls. T cell exosome-derived miR-142-3p acted as a pathogenic driver of immunopathology in SS. Exosomes containing miR-142-3p from activated T cells impaired the function of salivary gland epithelial cells. The mechanisms linked to epithelial secretory function, including Ca^2+^ flux, cAMP production, and protein secretion (66).

Type 1 diabetes (T1D) is an autoimmune disease initiated by the invasion of pancreatic islets by immune cells that selectively kill the β cells which is a hallmark of this disease. The autoimmune attack of β cells is a hallmark of T1D, but the detailed mechanisms remain poorly understood. Guay et al. provided evidence for the involvement of an exosome-mediated transfer of miR-142-3p, miR-142-5p, and miR-155 from T lymphocytes that trigger chemokines (CCL2, CCL7, and CXCL10) expression and apoptosis in recipient pancreatic β cells in T1D. On the other hand, inactivation of these miRNAs in recipient β cells prevents exosome-mediated apoptosis and protects non-obese diabetic (NOD) mice from diabetes development *in vivo* (67).

A better understanding of the mechanisms during germinal center (GC) reaction dynamics may supply new therapeutic strategies to modulate humoral responses (68). Transferred EV-miRNAs of T-cell origin target key genes to regulate B-cell function including antibody production and these small EVs is required for GC reaction (68). Immunological synapse (IS) is a highly specific means of intercellular communication between T cells and APCs during antigen recognition. Mittelbrunn et al. presented evidence that during cognate immune interactions there is a unidirectional transfer of miR-335 from T cell to APC in an Ag-specific manner (69). This genetic communication appears to connect with formation of the IS. Moreover, transferred miR-335 during immune synapse could down-regulate translation of SOX-4 mRNA in recipient cells (69).

Foxp3^+^ T regulatory (Treg) cells could prevent inflammatory damage, but the precise mechanisms are incompletely understood. In this regard, exosomal miRNA mediated autonomous gene silencing is a requirement for Treg cell-mediated suppression. Exosome-carried transfer of let-7d inhibited Th1 cell proliferation and IFN-γ secretion, which contributes to prevention and suppression of systemic disease (70).

In summary, the above studies reveal a mechanism of T cell mediated immunoregulation by miRNA-containing exosomes. Intriguingly, DCs derived exosomes could transfer functional miRNAs to target cells (52), whereas exosomes secreted by T cells transfer miRNA unidirectionally to DCs according to the previous reports (69). It suggests that exosomes generated from different cell types might execute their roles in distinctive manners.

## Interaction Between Exosomes, miRNAs and TLRs

**Figure 2:**
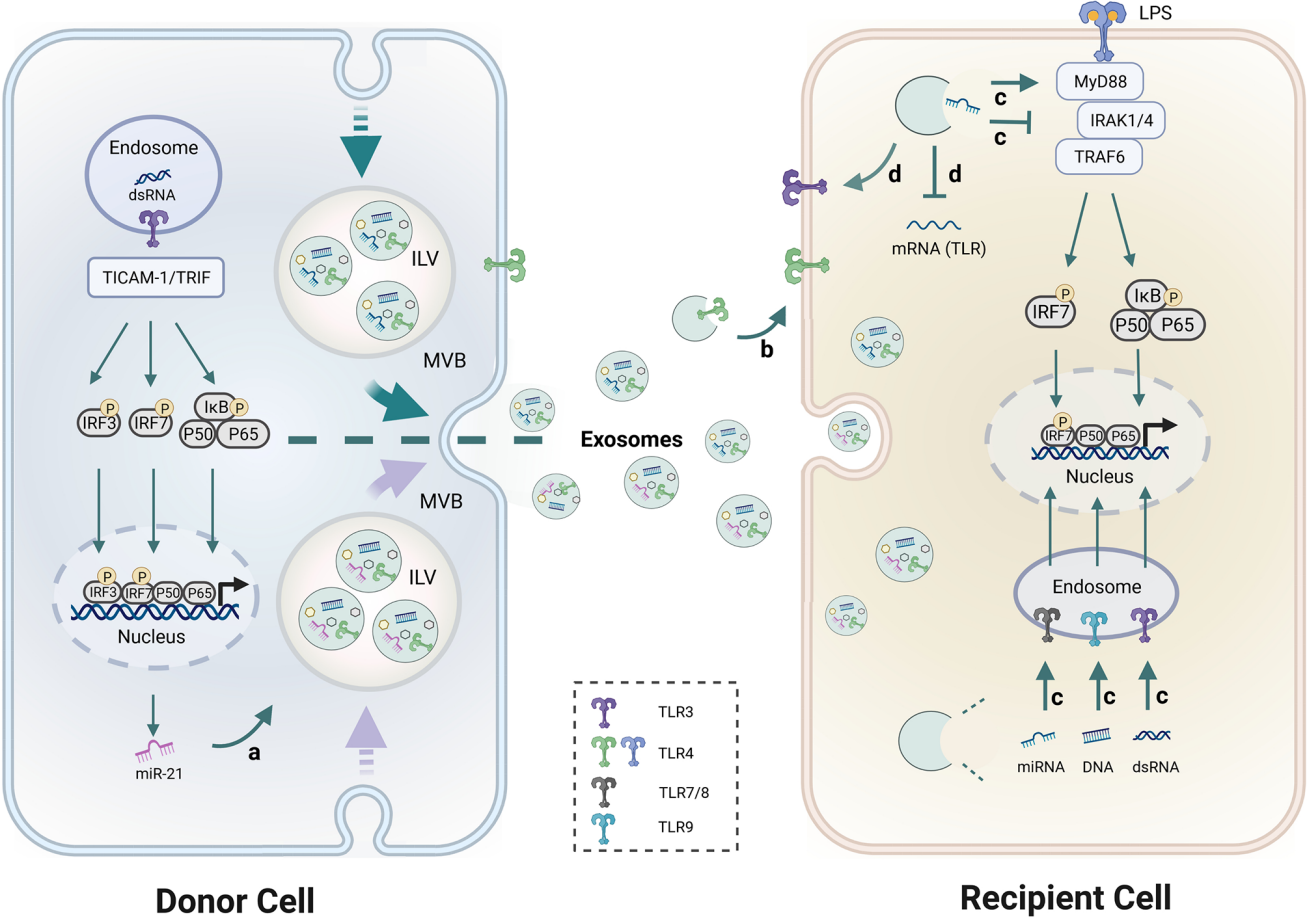
Schematic diagram of the interaction between exosomes, miRNAs and TLRs. a. TLR3 senses dsRNA and utilizes the adaptor TRIF to activate IRF3 and NF-κB. Activation of TLR3 and its adaptor TICAM-1 increased miR-21 levels in exosomes. b. TLRs like TLR4 within exosomes can be transferred from donor cells to recipient cells. c. Exosome-encapsulated miRNAs, dsRNA and DNAs have the ability to regulate TLR7/8, TLR3, and TLR9 signaling pathways respectively, and induce immune activation and immunosuppression. d. Exosomal-miRNAs were found to suppress or up-regulate TLRs expression. MyD88 and TRIF are two major TIR domain-containing adaptors downstream of TLRs. IRFs and NF-κB are the common downstream transcriptional factors in TLRs pathways that regulate gene expression. Detailed descriptions are provided in the main text.

TLRs recognize exogenous and endogenous stimuli to prime immune responses, and excessive activation of TLRs contributes to disease progression in several autoimmune disorders (79, 80). Lee et al. demonstrated that circulating exosomes from SLE could activate both surface and endosomal TLRs (10). Diverse biomolecules (proteins, RNAs and DNAs), especially miRNAs, in exosomes have been confirmed to be recognized by multiple TLRs and play a pivotal role in regulating inflammatory and immumodulatory effects on target cells (81, 82). Therefore, the relationship between miRNA, exosomes and TLRs was deemed important in discovering the mechanism of exosomal miRNAs in SLE progression. The interplay between exosomes, miRNAs, and TLRs occurs at different levels, which was shown as follows (**Figure 2**).

### TLRs Activation Regulates the Level of miRNAs in Exosomes

Activation of TLR3 and its adaptor TICAM-1 increased miR-21 levels in EVs but not intracellular miR-21 levels, which indicates a novel role of the TLR3/TICAM-1 pathway in controlling miR-21 levels in EVs. Consistently, the siRNA for TICAM-1 reduced miR-21 levels in EVs after stimulation of TLR3 suggesting that TICAM-1 augmented sorting of miR-21 to EVs (83). 25% of SLE patients have antiphospholipid antibodies (aPL) which are a heterogeneous population of autoantibodies that recognize anionic phospholipid-binding proteins. A previous study demonstrated that aPL upregulated levels of miR-146a-3p and miR-146a-5p in exosomes. This effect could be inhibited by LPS-RS meaning, which suggests that this up-regulation was TLR4-mediated (84).

### Exosomes Transfer TLRs to Recipient Cells

Exosomes mediate intercellular communication by transferring genomic materials between source and target cells. However, few studies have shown that TLRs within exosomes can be transferred to recipient cells. Zhang et al. Showed for the first time that TLR4 on wild type BMDC-derived exosomes can be taken up by TLR4 knockout BMDCs and increase cellular responsiveness to LPS by activating the TLR4/NF-κB pathway in recipient cells (85). These results indicate that functional TLR proteins themselves could also be transferred from original cells to recipient cells through exosome-like EVs.

### Exosomal-miRNAs Modulate TLR Activation

Zhao et al. demonstrated that exosomal miR-182 derived from MSC participated in the regulation of macrophage polarization by targeting TLR4/NF-κB signaling cascades (17). In addition, other miRNAs are also abundant in MSC derived exosomes that synergistically targeted the TLR4/NF-κB signaling pathway and significantly decreased the production of proinflammatory cytokines (16). A previous study found that exosome-delivered miR-548a-3p could regulate macrophage-mediated inflammatory response by targeting TLR4/NF-κB signaling pathway in RA (86). Exosomal miRNAs extracted from the plasma of SLE patients could induce the production of IFN-α by pDC which is a hallmark of SLE (87).

Exosome-delivered miRNAs could also act as endogenous ligands of human TLR7, and induce pDC activation in SLE patients (87). For instance, miR-21 affects the resolution of inflammation by interacting with TLR7/8 (82). miR-29b-containing exosomes participate in the regulation immune response *via* TLR7 signaling (88). miR-let-7b packaged in exosomes is a endogenous ligand of TLR7 which can bind to TLR7^+^ myeloid cells. Fusion of exosomal miR-let-7b into M2 cells differentiated these cells into M1 macrophages (89). The above studies suggest that exosomes-capsuled miRNAs have the ability to regulate specific TLRs signaling pathways and induce immune activation and immunosuppression.

### Exosome Carried miRNAs Regulate TLR Expression

Besides the regulating roles of exosomes in the downstream of TLRs signaling cascade, several studies have demonstrated the influence of exosomes on the expression of TLRs themselves. Exosomes derived from pancreatic cancer are enriched in miR-203 which down-regulates TLR4 expression and inhibits cytokines production (IL-12 and TNF-α) in DCs (90). miR-181c abrogates TLR4 expression by directly binding to its 3′-UTR to restrict the inflammatory response (91) which suggests that miR-181c is an essential therapeutic tool in anti-inflammation treatment. Moreover, exosomal-miR-181c from hUC-MSC inhibits the expression of TLR4 and subsequently reduces NF-κB/p65 activation (51). Similarly, Peng et al. identified that miR-216a-5p containing exosomes could also bind to 3′-UTR of TLR4 and suppress inflammatory response (92).

## Exosomal miRNAs as Potential Biomarkers for SLE

In clinical practice, diagnosis of SLE is complicated and challenging due to lack of ideal biomarkers with high specificity and sensitivity. Moreover, dysregulation of distinct miRNAs has been discussed as a pathogenic feature in autoimmune diseases like SLE (7, 93) and they are easily detectable in many biological fluids. Therefore, increasing number of studies focus on the effort to explore the potential of these molecules to serve as ideal biomarkers for SLE (**Table 2**).

**Table 2:** Altered expression and potential roles of exosomal miRNAs in renal damage.

Disease	Species	Source		Exo-miRNAs		Potential roles	Ref.
SLE/LN	Human	Urine		miR-146a ↑		Discriminating the presence of active LN	(9)
SLE/LN	Human	Urine		miR-146a↑		As a biomarker of albuminuria and disease flares in LN of SLE	(12)
LN	Human Mouse	Urine		miR-26a ↑		As a marker of injured podocytes in autoimmune glomerulonephritis	(94)
KD	Dog	Urine		miR-10a/b ↓		Reflecting the changes in renal functions and histopathology	(95)
LN	Human	Urine		let-7a ↓ miR-21 ↓		Guiding the clinical stage of LN patients	(96)
LN	Human	Urine		miR-29c ↓		Negative correlation with glomerular sclerosis	(13)
CKD	Human	Urine		miR-29c ↓		As a noninvasive marker for renal fibrosis	(97)
SLE/LN	Human	Urine		miR-150 ↑ miR-21 ↑ miR-29c ↓		For early diagnosis of kidney fibrosis in LN	(11)
LN	Human	Urine		miR-3135b ↑ miR-654-5p ↑ miR-146a-5p ↑		As novel non-invasive diagnostic markers for LNIV-CC	(98)
LN	Human	Urine		miR-31 ↑ miR-107 ↑ miR-146a-5p ↑		Promising markers for clinical outcomes	(99)
SLE	Human	Serum		miR-451a ↓		Serving as a potential biomarker and therapeutic target for SLE	(100)
SLE	Human	Serum		Hsa-miR-135b-5p ↑		As a promising diagnostic biomarker for SONFH in SLE	(101)

Exo, exosome; miR, microRNA; SLE, systemic lupus erythematosus; LN, lupus nephritis; KD, kidney disease; CKD, chronic kidney disease; LNIV-CC, type IV lupus nephritis with cellular crescent; SONFH, steroid-induced osteonecrosis of femoral head.

LN is characterized by autoantibody-induced renal damage and it is still a major cause of the morbidity and mortality in SLE. The current “gold standard” to predict renal outcome is renal biopsy, which is invasive and complicated. miRNAs are indispensable to regulate renal development, function and homeostasis processed in kidneys. It has been emphasized that there were several urinary miRNAs changes in LN. Following the discovery of urinary exosomes by Pisitkun et al. (102), there is great interest in using urinary exosomal-miRNAs as non-invasive biomarkers in renal diseases mainly owing to the easy collection and their effects in reflecting pathophysiological features of organism. Urinary miRNAs are primarily enriched in exosomes in SLE, and the increment was found in active LN (9).

miR-146a has been demonstrated to involve in kidney injury in murine lupus model and human lupus. Previous studies reported that urinary exosomal miR-146a was strongly enhanced in patients with SLE when compared to healthy controls (9, 12). And miR-146a enrichment in urinary exosomes from SLE was closely associated with renal damage index such as proteinuria, histological features and lupus activity suggesting a crucial role of miR-146a during the development of LN (12). Moreover, exosomal miR-146a was found to discriminate patients with active from inactive LN patients (9, 12) because the levels of exosomal miR-146a are highest in active LN patients who suffer from more severe kidney injury, such as increased glomerular sclerosis, tubular atrophy, and interstitial fibrosis (12, 98).

In addition, an increased level of miR-26a in urinary exosomes was found from patients of autoimmune glomerulonephritis, which is closely correlated with podocyte injuries indicating that altered miR-26a levels in exosomes may serve as a biomarker of injured podocytes in these patients (94).Urinary exosome-associated miRNAs act as epigenetic factors playing a crucial role in maintaining renal tubular cells in a paracrine man¬ner. In this sense, decreased miR-26a and -10a/b could reflect the changes in renal functions and histopathology in dogs (95).

Immune complexes mediated inflammation at resident kidney cells contributes to LN pathogenesis. Abnormal expression of miR-10a, -10b and let-7a could reflex the inflammatory phenotypes of renal cells because their expression was rapidly changed during the attack of resident kidney cells by acute immune complexes (103). Let-7a was demonstrated to be able to control inflammation *via* inhibiting IL-6 expression in spontaneous developed LN mouse model (104). Levels of urinary exosome-associated miR-21 and let-7a was significantly decreased in active LN patients and they were closely correlated with the clinical stage of LN (96). miR-21 also mediated anti-inflammatory effects by targeting several inflammatory molecules such as IL-12, TNF and IFN-γ which are associated with development of autoimmune disease (105). In addition, miR-21 in urine acts as a promising biomarker for kidney fibrosis and injuries (106, 107).

The expression levels of exosomal miRNAs in urine from chronic kidney disease (CKD) patients were significantly dysregulated compared to the healthy controls (108). Solé et al. demonstrated that miR-29c serves as a sensitive and specific biomarker for determining the degree of chronicity in LN because the decreased expression of miR-29c negatively correlated with glomerular sclerosis and histological chronicity index (13). A study in CKD patients indicated that the level of miR-29c in exosomes specifically correlated with both estimated glomerular filtration rate and degree of tubulointerstitial fibrosis, which suggests that exosomal miR-291c is a noninvasive biomarker for kidney fibrosis (97).

There is also an attractive method to use combinatory exosomal miRNAs as diagnosis of SLE/LN. For example, Solé et al. demonstrated that a urinary exosomal multimarker panel composed of miR-21, miR-29c, and miR-150 provides an effective strategy to detect early renal fibrosis, which could be used to predict disease progression in LN (11). Type IV lupus nephritis (LNIV) is the class of LN which is characterized by diffuse proliferative lesions. And the prognosis is worse in LNIV patients complicated by cellular crescent (LNIV-CC). By comparing miRNA expression profile in urinary exosomes between LNIV and LNIV-CC, Li et al. found that LNIV-CC shows a unique expression profile of miRNAs. Among them, miR-146a-5p, miR-654-5p, and miR-3135b in urinary exosomes possess predictive values for LNIV-CC (98). Another set of urinary exosomal miRNA expression profile was identified to evaluate clinical outcomes in patients with LN following conventional therapy. It was demonstrated that the levels of miR-135b-5p, miR-107, and miR-31in urinary exosomes were up-regulated in responder patients, and they can be used as early markers of LN outcomes. Moreover, they contribute to renal recovery by inhibiting HIF-1α (99).

**Figure 3:**
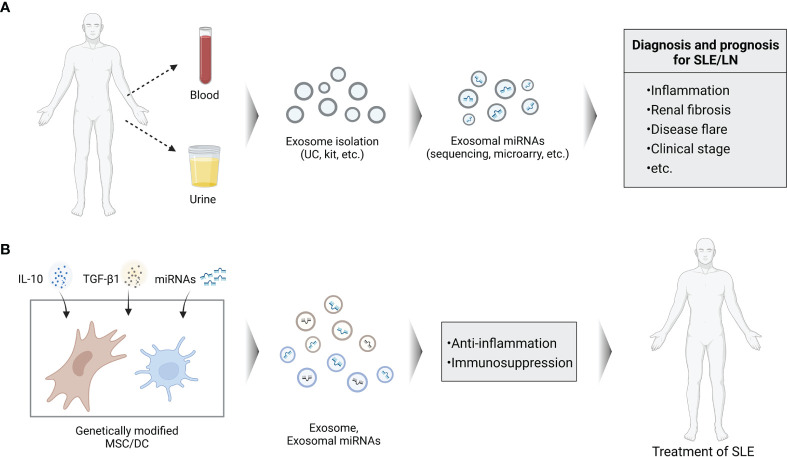
Schema of potential roles of exosomal miRNAs in SLE. **(A)** Analysis of exosomal miRNAs isolated from blood or urine can be used for the clinical diagnosis and prognosis of SLE. **(B)** Genetically modified cells to secrete exosomes containing specific miRNAs can be used as cell-free agents of SLE.

A recent study from Tan et al. showed that downregulated exosomal miR-451a in serum is correlated with SLE disease activity and renal damage. In addition, serum exosomes could transfer miR-451a into B cells and CD4^+^ T cells, indicating their crucial roles in intercellular communication (100). The risk of steroid-induced osteonecrosis of femoral head (SONFH) in patients with SLE is 100 times higher compared to the general population (109). Zhang et al. demonstrated that exosomal has-miR-135b-5p in serum could act as unique diagnostic biomarkers for SONFH in SLE patients by providing experimental evidence that specific exosome-associated has-miR-135b-5p is differentially expressed between healthy individuals and SLE patients (101). Based on the above findings, exosome-associated miRNAs in biological fluids especially in the urine could act as a useful, non-invasive biomarkers for diagnosis and prognosis of SLE/LN in the clinical setting (**Figure 3**).

## Exosomal-miRNAs as Therapeutic Potential for SLE

Although some anti-miRNAs and miRNA mimics have successfully entered Phase I and II clinical trials, an optimal delivery system with minimal toxicity and lower immunogenicity, as well as more specific target effects are urgently needed during the exploring of miRNA-based therapy. As a novel delivery system, exosomes possess multiple advantages compared to current miRNA delivery vehicles. Due to the protection of their unique structure, miRNA contents are remarkably stable in exosomes and resistant to degrading conditions (52, 110). Exosomes are able to travel across the blood-brain barrier (BBB) and have low immunogenicity which also define them as ideal therapeutic vehicles. Exosomes generated *in vitro* by miRNA transfected cells could efficiently deliver these miRNAs to recipient cells achieving the desired effects showing their intrinsic targeting activity (111). Therefore, patients’ own exosomes can be isolated and modified by specific therapeutic agents like miRNA. These engineered exosomes can internalize into the target cells with less immunogenic than other foreign delivery vectors (112). Given the immunomodulatory properties of exosomal miRNAs, there is growing interest by using exosomal miRNAs as therapeutic approach. Exosomes derived from MSCs and DCs hold a promising therapeutic approach as cell-free agents to treat autoimmune disorders owing to their pivotal immunomodulatory roles (**Figure 3**).

Numerous studies have found that both MSCs and MSC-derived exosomes exhibit significantly therapeutic effects in the treatment of kidney injury (113–115). MSC-derived exosomes have a similar miRNA profile as their origin cells, which may explain their therapeutic mechanisms (116). In some cases, exosomes may be more advantageous than their parental cells, including easier storage, greater half-life, and increased stability (117). MSC transplantation has been regarded as a safe and effective therapeutic approach in SLE while these MSCs exhibited senescent characteristics. Reversing the senescent phenotypes of MSCs could improve therapeutic effects of autologous MSCs transplantation in SLE (118). Levels of miR-146a in the serum exosomes of SLE patients are significantly declined compared with healthy group. These exosomes encapsuled miR-146a was internalized into MSCs and alleviate the senescence of MSCs by targeting TRAF6/NF-κB pathway (119).

In this sense, previous studies investigated the therapeutic potential of DC-derived exosomes in autoimmune diseases. For instance, exosomes secreted from IL-10 treated DCs (120), or genetically modified IL-4 expressing DCs (121) possess immunosuppressive and anti-inflammatory properties. Administration of these exosomes was able to suppress murine CIA as well as reduce severity of established arthritis (120, 121). Similarly, injection of TGF-β1 modified DCs was demonstrated to reduce disease activity in murine inflammatory bowel disease (IBD) (122). Thus, genetic modification of exosomes to express specific molecule, can enhance their ability in suppression of inflammatory and may constitute a novel type cell-free system for the treatment of autoimmune disorders. Nevertheless, the use of exosomes/exosomal-miRNAs for therapy is challenging. Until now, studies on DCs derived exosomes carrying miRNA to treat autoimmune diseases are very rare. Compared to their applications in cancer, less information is available on the role of exosomal-miRNAs in the pathogenesis and treatment of SLE.

## Conclusion

This review has summarized the characteristics of exosomes, the mechanisms of sorting miRNAs into exosomes, and the immunomodulatory effects of exosomal-miRNAs. A better and more complete understanding of various forms of immune cells derived exosomal miRNAs is necessary for the development of new and more effective strategies for optimizing SLE therapies in the future. Abnormal expression levels of exosomal-miRNAs may serve as ideal diagnostic biomarkers in renal injury of SLE, which is also discussed. Furthermore, this review provides new insights into a complex interplay between exosomes, miRNAs and TLRs, which might provide promising therapeutic targets for SLE.

Our understanding of the biology of exosomal-miRNAs increased markedly, but it is still in an early stage and several challenges remain to overcome. Further researches are required to decipher the molecular mechanisms involved in exosomes biogenesis, cargo release and target specificities. For instance, 1) an effective and standard way to generate most efficient and pure exosomes remains to be elucidated; 2) more precise mechanisms underlying complex components and immunoregulatory functions of exosomal-miRNAs are required; 3) sensitive and accurate miRNA analysis is necessary; 4) more stable and better therapeutic effects of exosomal-miRNAs *in vivo* need further exploration. After solving these basic and clinical problems, exosomes may be applied in autoimmune diseases in the future.

In summary, although the research on application of exosomes still face several problems, the advantages and potential application of exosomal-miRNAs are attracting increasing attention. Intensive investigation on the biological functions and the molecular mechanisms of exosomal-miRNAs involved in SLE will provide potential biomarkers and novel therapeutic strategies and facilitate the clinical translation of exosomes.

## Author Contributions

WW and CH conceived and designed the review outline. All authors contributed to the writing and critical review of the manuscript. CH and WW acquired the funding support.

## Funding

This work was supported by grants from the National Natural Science Foundation of China [81901660, 81802963, 81972261]; China Scholarship Council [201808330646]; Zhejiang Provincial Natural Science Foundation of China [LY18H160046]; and Zhejiang Medical Science Foundation [2018KY531]; and Lin He’s New Medicine and Clinical Translation Academician Workstation Research Fund (18331215).

## Conflict of Interest

The authors declare that the research was conducted in the absence of any commercial or financial relationships that could be construed as a potential conflict of interest.

## Publisher’s Note

All claims expressed in this article are solely those of the authors and do not necessarily represent those of their affiliated organizations, or those of the publisher, the editors and the reviewers. Any product that may be evaluated in this article, or claim that may be made by its manufacturer, is not guaranteed or endorsed by the publisher.
